# Comparison of maternal and neonatal outcomes of COVID-19 before and after SARS-CoV-2 omicron emergence in maternity facilities in Malawi (MATSurvey): data from a national maternal surveillance platform

**DOI:** 10.1016/S2214-109X(22)00359-X

**Published:** 2022-09-22

**Authors:** Leonard Mndala, Edward J M Monk, Deborah Phiri, Jennifer Riches, Regina Makuluni, Luis Gadama, Fannie Kachale, Rosemary Bilesi, Malangizo Mbewe, Andrew Likaka, Chikondi Chapuma, Moses Kumwenda, Bertha Maseko, Chifundo Ndamala, Annie Kuyere, Laura Munthali, Marc Y R Henrion, Clemens Masesa, David Lissauer

**Affiliations:** a Malawi–Liverpool–Wellcome Clinical Research Programme, Blantyre, Malawi; b Kamuzu University of Health Sciences, Blantyre, Malawi; c Ministry of Health, Lilongwe, Malawi; d Malawi Blood Transfusion Services, Blantyre, Malawi; e Universidade Federal de Pernambuco, Recife, Brazil; f Liverpool School of Tropical Medicine, Liverpool, UK; g University of Liverpool, Liverpool, UK

## Abstract

**Background:**

Outcomes of omicron-associated COVID-19 in pregnancy have not been reported from low-resource settings, and data from sub-Saharan Africa before the emergence of omicron are scarce. Using a national maternal surveillance platform (MATSurvey), we aimed to compare maternal and neonatal outcomes of COVID-19 in Malawi during the omicron wave to the preceding waves of beta and delta.

**Methods:**

All pregnant and recently pregnant patients, up to 42 days following delivery, admitted to 33 health-care facilities throughout Malawi with symptomatic, test-proven COVID-19 during the second (beta [B.1.351]: January to April, 2021), third (delta [B.1.617.2]: June to October, 2021), and fourth (omicron [B.1.1.529]: December 2021 to March, 2022) waves were included, with no age restrictions. Demographic and clinical features, maternal outcomes of interest (severe maternal outcome [a composite of maternal near-miss events and maternal deaths] and maternal death), and neonatal outcomes of interest (stillbirth and death during maternal stay in the health-care facility of enrolment) were compared between the fourth wave and the second and third waves using Fisher's exact test. Adjusted odds ratios (ORs) for maternal outcomes were estimated using mixed-effects logistic regression.

**Findings:**

Between Jan 1, 2021, and March 31, 2022, 437 patients admitted to 28 health-care facilities conducting MATSurvey had symptoms of COVID-19. SARS-CoV-2 infection was confirmed in 261 patients; of whom 76 (29%) had a severe maternal outcome and 45 (17%) died. These two outcomes were less common during the fourth wave (omicron dominance) than the second wave (adjusted OR of severe maternal outcome: 3·96 [95% CI 1·22–12·83], p=0·022; adjusted OR of maternal death: 5·65 [1·54–20·69], p=0·0090) and the third wave (adjusted OR: 3·18 [1·03–9·80], p=0·044; adjusted OR: 3·52 [0·98–12·60], p=0·053). Shortness of breath was the only symptom associated with poor maternal outcomes of interest (p<0·0001), and was less frequently reported in the fourth wave (23%) than in the second wave (51%; p=0·0007) or third wave (50%; p=0·0004). The demographic characteristics and medical histories of patients were similar across the three waves. During the second and third waves, 12 (13%) of 92 singleton neonates were stillborn or died during maternal stay in the health-care facility of enrolment, compared with 0 of the 25 born in the fourth wave (p=0·067 *vs* preceding waves combined).

**Interpretation:**

Maternal and neonatal outcomes from COVID-19 were less severe during the fourth wave of the SARS-CoV-2 pandemic in Malawi, during omicron dominance, than during the preceding beta and delta waves.

**Funding:**

Bill & Melinda Gates Foundation, Wellcome Trust, and the National Institute for Health and Care Research.

**Translation:**

For the Chichewa translation of the abstract see Supplementary Materials section.

## Introduction

COVID-19 has been shown by a large number of studies to increase the risk of maternal and neonatal morbidity and mortality.^1^ However, very little is known about the impact of COVID-19 on maternal health outcomes in sub-Saharan Africa.^2^, ^3^, ^4^, ^5^, ^6^, ^7^, ^8^ Although it is likely that COVID-19 outcomes in pregnancy differ between global regions—due to differences in health-care systems, resources, and prevalence of background disease—data from low-resource settings are scarce. One of the most robust systematic reviews reporting maternal and neonatal outcomes for COVID-19 in pregnancy, updated in late May, 2022, included extremely few studies from sub-Saharan Africa or low-resource settings, despite comprising data from 435 studies.^1^

**Research in context**

**Evidence before this study**

A living systematic review by Allotey and colleagues, most recently updated on May 30, 2022, included 435 studies and reported that pregnant and recently pregnant women with proven SARS-CoV-2 infection had an odds ratio of mortality that was 6·09 times higher than those without (95% CI 1·82–20·38). It also reported poorer neonatal outcomes. Allotey and colleagues described a shift in geographical reporting of COVID-19 outcomes in pregnancy since their previous update in October, 2020, but noted that there remain very few reports from Africa. INTERCOVID, a multinational maternal and neonatal morbidity and mortality study that included data from Ghana and Nigeria, reported a relative risk of maternal death of 22·26 (95% CI 2·88–172·11) in patients with COVID-19, compared with those without. Given the emergence of the omicron SARS-CoV-2 variant, which is reported to have different symptom and virulence profiles to wild-type SARS-CoV-2 and previously circulating variants of concern, the clinical presentations in maternity settings and the rates of adverse maternal and neonatal outcomes due to COVID-19 are likely to have changed since Allotey and colleagues' latest update (search run on April 27, 2021). We searched titles and abstracts in PubMed, Embase, and Europe PMC's preprint database on July 2, 2022 for articles published in any language since Nov 24, 2021 using the following search terms (MeSH terms limited to PubMed): ((COVID*) OR (SARS-CoV-2) OR (coronavirus) OR (COVID-19 [MeSH term])) AND ((pregnan*) OR (matern*) OR (labor) OR (labour) OR (birth*) OR (pregnancy [MeSH term])) AND ((omicron) OR (B.1.1.529) OR (BA.1) OR (BA.2)). Full-text manuscripts were obtained for all 28 articles that met our search criteria; six reported the demographic and clinical characteristics of cases in maternity settings (data from India, Ireland, Türkiye, the UK, and the USA) and three compared outcomes between periods of omicron dominance and pre-omicron (data from India, Türkiye, and the USA). In two of these high-resource settings (India and the USA), the unadjusted odds ratio of severe or critical illness in pregnant women with COVID-19 was 5–10 times smaller during periods of omicron dominance than during a period before delta dominance. In the third setting (Turkiye), there were insufficient data from the omicron wave to make any meaningful comparisons.

**Added value of this study**

Using a national reporting system (MATSurvey), we present the demographic and clinical characteristics of pregnant and recently pregnant women admitted to maternity inpatient settings across Malawi throughout the second, third, and fourth waves of the SARS-CoV-2 pandemic (January 2021–March 2022), and report both maternal and neonatal outcomes. Our study adds to the scarce data on maternal COVID-19 in sub-Saharan Africa, and provides the first characterisation of maternal COVID-19 in a low-resource setting since omicron circulation and dominance. We found lower rates of severe maternal outcome, maternal death, and adverse neonatal outcomes (stillbirth or death during maternal stay in the health-care facility of enrolment) in Malawi's fourth wave of SARS-CoV-2 (when omicron was dominant) than in the second and third waves, when beta and delta, respectively, were dominant.

**Implications of all the available evidence**

Maternal and neonatal outcomes of COVID-19 during the fourth wave of SARS-CoV-2 circulation, with omicron variant dominance, in Malawi appeared better than during previous waves. Our findings are crucial to understanding the evolving global burden of COVID-19 in a population with a high level of background maternal and neonatal morbidity and mortality.

Since its emergence in November, 2021, the omicron variant (B.1.1.529) of SARS-CoV-2 has become the variant of circulating dominance globally. Less virulent than previous variants of concern and wild-type SARS-CoV-2,^9^, ^10^, ^11^ it is likely that omicron infection in pregnancy has different presentations and outcomes than those reported for preceding variants of concern.^12^, ^13^, ^14^, ^15^, ^16^, ^17^ However, to our knowledge at the time of writing, there are no published or pre-print manuscripts that either characterise the clinical presentation or report the clinical outcomes of COVID-19 in pregnancy during periods of omicron dominance from low-resource settings.

To understand and mitigate the effects of COVID-19 in pregnant and recently pregnant (≤42 days post-birth) patients, including patients during periods of antenatal, peripartum, post-partum, and post-abortion care, Malawi's Ministry of Health collaborated with the Malawi–Liverpool–Wellcome Clinical Research Programme to implement an online maternal COVID-19 surveillance platform (MATSurvey). MATSurvey was implemented across all district and central hospitals in the country. Here, we aimed to consider the demographic characteristics, clinical manifestations, and maternal and neonatal outcomes of pregnant and recently pregnant patients admitted to all district and central hospitals in Malawi with symptoms of COVID-19 and confirmed SARS-CoV-2 infection. We contrasted data from the second (with beta [B.1.351] dominance) and third (with delta [B.1.617.2] dominance) waves of the SARS-CoV-2 pandemic with the fourth (with omicron dominance).

## Methods

### Study design and participants

MATSurvey utilises data from 33 sites across Malawi, comprising all four Central Hospitals (Queen Elizabeth Central Hospital in Blantyre, Kamuzu Central Hospital in Lilongwe, Mzuzu Central Hospital in Mzuzu, and Zomba Central Hospital in Zomba), the two District Health Offices of Blantyre and Zomba, and all of Malawi's 27 district hospitals.^18^

Eligible participants were pregnant and recently pregnant women, enrolled up to 42 days following delivery, who were admitted to health-care facilities within the catchment areas of participating hospitals and District Health Offices, providing national coverage. There were no age restrictions. We included women during periods of antenatal, peripartum, postpartum, and post-abortion care. Inclusion criteria were symptoms suggestive of COVID-19 (fever, cough, shortness of breath, arthralgia, fatigue, gastrointestinal symptoms, headache, loss of taste or smell, rhinorrhoea, or sore throat) and a positive SARS-CoV-2 PCR or lateral flow device test result on admission to or during their stay at a health-care facility. Asymptomatic patients were not included, and very rarely underwent SARS-CoV-2 testing in clinical settings.

The observation period of this study was Jan 1, 2021 to March 31, 2022, and contained the second, third, and fourth waves of the SARS-CoV-2 pandemic in Malawi. We used the SARS-CoV-2 variants circulating in Malawi during these periods, established previously,^19^, ^20^ to define these waves according to circulating variants of dominance: beta in the second wave (Jan 1–April 30, 2021), delta in the third wave (June 1–Oct 31, 2021), and omicron in the fourth wave (Dec 1, 2021–March 31, 2022). May and November, 2021 were not included, to separate waves over periods of very low transmission, during which there were changes in variant of dominance.

Data for this analysis were entirely anonymised and made available to the authors by the authorisation of the Malawi Ministry of Health and College of Medicine Research Ethics committee. Patients' confidentiality, and adherence to the committee's ethical requirements was maintained throughout all stages of analysis.

### Data sources

MATSurvey is a national maternal surveillance platform that records all severe maternal outcomes (maternal near-miss events and maternal deaths), according to adapted WHO definitions. Roll-out was expedited nationally during Malawi's first wave of the SARS-CoV-2 pandemic in July, 2020, with additional surveillance of all inpatients clinically suspected to have COVID-19, regardless of disease severity.

Data were uploaded to the MATSurvey platform using mobile data collection tools by safe motherhood coordinators at each study site, throughout the observation period (OpenDataKit version 1.21.0). Safe motherhood coordinators are nurse-midwives, trained by the Ministry of Health in active case-finding and data collection, responsible for recording routine data from clinical notes, handover files, and hospital registers on a daily basis. With the introduction of the MATSurvey platform, safe motherhood coordinators were further trained in digital data collection and entry, using a bespoke tablet application developed by the Malawi–Liverpool–Wellcome Clinical Research Programme, in collaboration with the Ministry of Health, to enhance the surveillance potential of these routinely collected data. Data capture forms consisted of an initial enrolment survey, followed by a daily survey during the participants' stay in the health-care facility to capture evolving outcomes. To ensure the reliability of uploaded data and address inconsistencies in data entry, validation rules were included (skip-logics and cross-validation against a second entry).

Safe motherhood coordinators at Central Hospitals reported all cases of suspected and proven COVID-19 that occurred within their facility through active case-finding, conducted in all clinical areas that could admit patients who met our inclusion criteria. Safe motherhood coordinators at District Health Offices and District Hospitals reported all cases from within their facility using the same methods, as well as from primary maternity services with inpatient capacity at the district level throughout Malawi.

### Outcomes

We considered both maternal and neonatal outcomes up until the point of maternal discharge from the health-care facility of enrolment. Maternal outcomes of interest were severe maternal outcome and maternal death. Severe maternal outcome was defined as a composite of maternal near-miss events and maternal deaths, and maternal near-misses were established from a prespecified list of criteria in pregnancy or recent pregnancy, signifying that a patient almost died (ie, developed life-threatening organ dysfunction).^21^ Neonatal outcomes of interest were stillbirth and death during maternal stay in the health-care facility of enrolment.

### Statistical analysis

Weekly admission to participating health-care facilities, maternal outcome, and neonatal outcome counts were calculated for the duration of the observation period and presented as stacked histograms. The demographic characteristics, medical histories, and symptoms of participants admitted during each wave were established, and the proportions from the fourth wave were compared with those of the second and third waves using Fisher's exact test.

Mixed-effects logistic regression analyses were performed to investigate the associations between the SARS-CoV-2 wave at admission (to the health-care facility of enrolment) and each maternal outcome of interest (severe maternal outcome and maternal death); patient characteristics (age group, presence of comorbidities, HIV status, and parity group [number of pregnancies >28 weeks' gestation]) were considered for inclusion as categorical variables (fixed effects) within facility-level clusters (random effects). Initially, the adjusted odds ratios (ORs), 95% CIs and associated Wald χ^2^ test p values for each characteristic were calculated, adjusting for facility only. A multivariable mixed-effects logistic regression model was then built to explore the association between the SARS-CoV-2 wave at admission and each maternal outcome of interest; after a conceptual model exercise, age group, HIV status, and parity group were each considered for inclusion in the models as a potential confounder (parity was included as having children can affect mixing patterns). Presence of comorbidity (excluding HIV) was not regarded as conceptually associative with SARS-CoV-2 wave, and therefore not considered further.

Additional analyses included estimating the crude association between symptoms and maternal outcomes of interest (unadjusted OR, 95% CI and associated χ^2^ p values) and comparing the adverse neonatal outcomes for singleton pregnancies between the fourth wave and preceding waves (Fisher's exact test).

Analyses were performed using Stata (version 15.1).

### Role of the funding source

The funders had no role in study design, data collection, data analysis, data interpretation, writing of the report, or the decision to submit this paper for publication.

## Results

**Figure 1:**
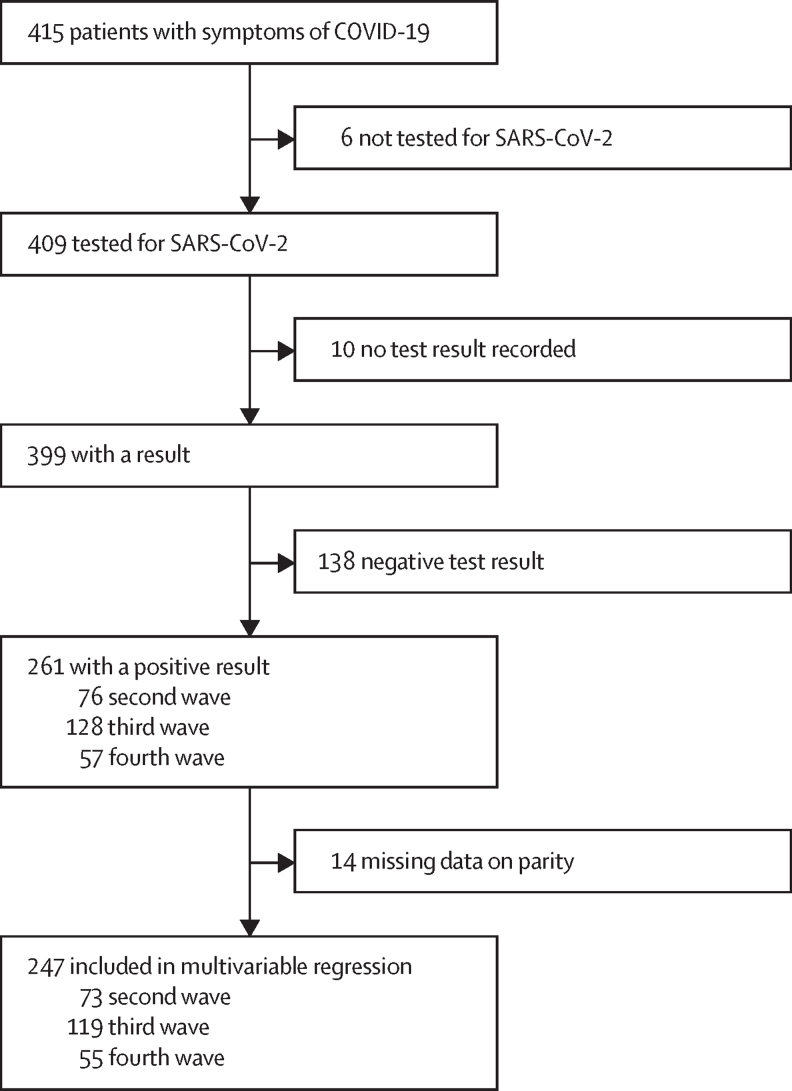
Study profile

Between Jan 1, 2021, and March 31, 2022, 437 patients admitted to 28 facilities conducting MATSurvey had symptoms of COVID-19. Of these, 22 (5%) of 437 were excluded as they were admitted in May or November, 2021. Confirmatory SARS-CoV-2 tests were recorded as having taken place in 409 (99%) of the 415 remaining patients: 373 (90%) of 415 received a PCR test, with or without a lateral flow device, and 36 (9%) of 415 were tested via a lateral flow device only. Of these, 399 (98%) of 409 had results recorded. Overall, SARS-CoV-2 infection was confirmed in 261 (63%) of the 415 symptomatic patients: 76 (60%) of 126 in the second wave, 128 (70%) of 182 in the third, and 57 (53%) of 107 in the fourth (figure 1).

Of the 261 individuals with symptomatic SARS-CoV-2 infection, 14 (5%) had missing data (parity only; table 1). The demographic characteristics and medical histories of patients in the fourth wave were similar to those in the second and third waves. Most patients in each wave were in the 20–34-years age group (180 [69%] of 261). The majority of patients had an unremarkable medical history, with 230 (88%) having no documented comorbidities (excluding HIV). HIV was recorded in 25 (10%) patients across the three waves.

**Table 1:** Demographic characteristics and medical histories of participants, by pandemic wave, in Malawi

		**Second wave (n=76), n (%)**	**Third wave (n=128), n (%)**	**Fourth wave (n=57), n (%)**	**Wave comparison, p value**	
					Second *vs* fourth	Third *vs* fourth
Age, years						
	<20	16 (21%)	13 (10%)	11 (19%)	0·831	0·100
	20–34	50 (66%)	90 (70%)	40 (70%)	0·708	1·000
	≥35	10 (13%)	25 (20%)	6 (11%)	0·790	0·142
Comorbidities						
	At least one comorbidity	6 (8%)	17 (13%)	8 (14%)	0·269	1·000
	Asthma	3 (4%)	7 (5%)	4 (7%)	..	..
	Diabetes	0	1 (1%)	0	..	..
	Heart disease	1 (1%)	11 (1%)	0	..	..
	Hypertension	1 (1%)	11 (9%)	4 (7%)	..	..
	Renal disease	1 (1%)	1 (1%)	0	..	..
HIV status						
	HIV positive	3 (4%)	12 (9%)	10 (18%)	0·016	0·140
Parity (>28 weeks)						
	Nulliparous	34 (45%)	48 (38%)	20 (35%)	0·288	0·869
	1–3	35 (46%)	53 (41%)	31 (54%)	0·383	0·112
	≥4	4 (5%)	18 (14%)	4 (7%)	0·724	0·222
	Missing data	3 (4%)	9 (7%)	2 (4%)	1·000	0·507

p values for the wave comparison were calculated via Fisher's exact test.

Fever was recorded in a similar proportion of patients in the fourth wave as in the second and third waves (table 2). Cough was less commonly reported in the fourth wave than in the third wave, but it was similarly reported in the fourth and second waves. Shortness of breath was less common in the fourth wave than in the second and third waves (23% in the fourth wave *vs* 51% in the second wave [p=0·0007] and 50% in the third wave [p=0·0004]).

**Table 2:** Symptoms of participants with confirmed COVID-19, by pandemic wave, in Malawi

	**Second wave (n=76), n (%)**	**Third wave (n=128), n (%)**	**Fourth wave (n=57), n (%)**	**Wave comparison, p value**	
				Second *vs* fourth	Third *vs* fourth
Fever	41 (54%)	57 (45%)	26 (46%)	0·383	1·000
Cough	54 (71%)	117 (91%)	35 (61%)	0·268	<0·0001
Shortness of breath	39 (51%)	64 (50%)	13 (23%)	0·0007	0·0004
Fever, cough, or shortness of breath	75 (99%)	127 (98%)	48 (84%)	0·0022	0·0001
Arthralgia	11 (14%)	11 (9%)	4 (7%)	0·268	1·000
Fatigue	11 (14%)	8 (6%)	2 (4%)	0·041	0·726
Gastrointestinal symptoms (diarrhoea or vomiting)	5 (7%)	10 (8%)	6 (11%)	0·529	0·576
Headache	18 (24%)	23 (18%)	13 (23%)	1·000	0·430
Loss of taste or smell	7 (9%)	13 (10%)	5 (9%)	1·000	1·000
Rhinorrhoea	4 (5%)	10 (8%)	5 (9%)	0·494	0·779
Sore throat	6 (8%)	12 (9%)	7 (12%)	0·557	0·602

p values for the wave comparison were calculated via Fisher's exact test.

**Figure 2:**
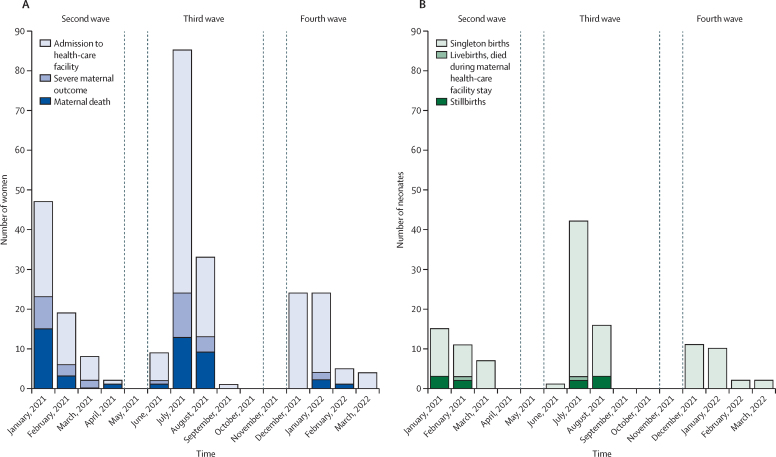
Monthly number of maternal and neonatal adverse outcomes during the second, third, and fourth waves of the SARS-CoV-2 pandemic in Malawi (A) Total maternal admissions with overlay counts of severe maternal outcomes and maternal deaths. (B) Total number of neonates born (singleton pregnancies) with overlay counts of deaths during maternal stay in the health-care facility of enrolment (if liveborn) and stillbirths. Dotted lines denote months that were not included to separate waves.

Throughout the observation period, 76 (29%) of the 261 participants had a severe maternal outcome recorded and 45 (17%) died (figure 2A). Both severe maternal outcome and maternal death were less frequently observed in the fourth wave (severe maternal outcome in five [9%] of 57 patients, maternal death in three [5%]) than in the second wave (severe maternal outcome in 32 [42%] of 76 patients, maternal death in 19 [25%]) and the third wave (severe maternal outcome in 39 [30%] of 128 patients, maternal death in 23 [18%]). There were no severe maternal outcomes or maternal deaths in the 11 patients that did not have fever, cough, or shortness of breath throughout the observation period.

The adjusted ORs of severe maternal outcome and maternal death according to SARS-CoV-2 wave at admission are shown in table 3 and table 4, respectively. Adjustment for facility only (unadjusted for patient characteristics) showed higher odds of severe maternal outcome and maternal death in the second and third waves than in the fourth wave (second *vs* fourth wave: severe maternal outcome adjusted OR 4·40 [95% CI 1·43–13·54, p=0·0097] and maternal death adjusted OR 6·00 [1·68–21·43, p=0·0058]); third *vs* fourth wave: severe maternal outcome adjusted OR 3·55 [1·22–10·29, p=0·020] and maternal death adjusted OR 3·94 [1·13–13·72, p=0·031]). These associations remained after adjustment for facility and patient age, HIV status, and parity (second *vs* fourth wave: severe maternal outcome adjusted OR 3·96 [1·22–12·83, p=0·022] and maternal death adjusted OR 5·65 [1·54–20·69, p=0·0090]); third *vs* fourth wave: severe maternal outcome adjusted OR 3·18 [1·03–9·80, p=0·044] and maternal death adjusted OR 3·52 [0·98–12·60, p=0·053]). There were no demographic subgroups associated with severe maternal outcome or maternal death.

**Table 3:** Associations of SARS-CoV-2 wave, age, HIV status, and parity with severe maternal outcome, unadjusted and adjusted for patient characteristics

	OR, adjusted for facility only*(95% CI)	**p value**	**OR, adjusted for facility and patient characteristics**†**(95% CI)**	**p value**
**SARS-CoV-2 wave**				
Second wave	4·40 (1·43–13·54)	0·0097	3·96 (1·22–12·83)	0·022
Third wave	3·55 (1·22–10·29)	0·020	3·18 (1·03–9·80)	0·044
Fourth wave	1 (ref)	..	1 (ref)	..
**Age, years**				
<20	0·52 (0·29–1·29)	0·157	0·72 (0·25–2·09)	0·545
20–34	1 (ref)	..	1 (ref)	..
≥35	0·94 (1·64–4·83)	0·069	1·99 (0·78–5·04)	0·148
**HIV status**				
HIV positive	1·87 (0·68–5·18)	0·226	2·23 (0·71–7·00)	0·168
**Parity**				
Nulliparous	0·54 (0·27–1·08)	0·080	0·67 (0·30–1·47)	0·313
1–3	1 (ref)	..	1 (ref)	..
≥4	0·98 (0·35–2·77)	0·974	0·74 (0·23–2·32)	0·604

OR=odds ratio.

* Adjusted for facility (random effects).

† Adjusted for age group, parity group, and HIV status (fixed effects) and facility (random effects).

**Table 4:** Associations of SARS-CoV-2 wave, age, HIV status, and parity with maternal death, unadjusted and adjusted for patient characteristics

	OR, adjusted for facility only*(95% CI)	**p value**	**OR, adjusted for facility and patient characteristics**†**(95% CI)**	**p value**
**SARS-CoV-2 wave**				
Second wave	6·00 (1·68–21·43)	0·0058	5·65 (1·54–20·69)	0·0090
Third wave	3·94 (1·13–13·72)	0·031	3·52 (0·98–12·60)	0·053
Fourth wave	1 (ref)	..	1 (ref)	..
**Age, years**				
<20	0·61 (0·21–1·79)	0·370	0·87 (0·27–2·82)	0·821
20–34	1 (ref)	..	1 (ref)	..
≥35	1·74 (0·74–4·08)	0·204	1·69 (0·66–4·33)	0·270
**HIV status**				
HIV positive	0·73 (0·20–2·66)	0·635	0·82 (0·21–3·15)	0·768
**Parity**				
Nulliparous	0·64 (0·30–1·36)	0·246	0·68 (0·29–1·58)	0·373
1–3	1 (ref)	..	1 (ref)	..
≥4	1·07 (0·34–3·36)	0·904	0·78 (0·24–2·54)	0·677

OR=odds ratio.

* Adjusted for facility (random effects).

† Adjusted for age group, parity group, and HIV status (fixed effects), and facility (random effects).

The only symptom associated with poor maternal outcomes was shortness of breath; the unadjusted OR of severe maternal outcome was 9·61 (95% CI 4·62–19·96, p<0·0001) and maternal death was 8·02 (3·37–19·11, p<0·0001) compared with patients without shortness of breath.

During the wave observation periods, 134 neonates were born to 125 patients within our cohort, including seven sets of twins and one set of triplets. When considering only singleton births (n=117), there were 33 in the second wave, 59 in the third, and 25 in the fourth. In the second and third waves, 28 (85%) of 33 and 54 (92%) of 59 were livebirths respectively and, of these, 27 (96%) of 28 and 53 (98%) of 54 survived until maternal discharge from the health-care facility of enrolment (figure 2B). During the fourth wave, all 25 neonates born were livebirths and all survived until maternal discharge (p=0·067 when comparing adverse neonatal outcomes in the fourth wave with the second and third waves combined).

## Discussion

There is a paucity of data from low-resource settings on maternal and neonatal outcomes in pregnant and recently pregnant patients with COVID-19. Our results are a substantial contribution to this body of evidence, reporting data from a national surveillance system in Malawi during periods of SARS-CoV-2 beta, delta, and omicron variant dominance. We found that the second (beta) and third (delta) waves of the SARS-CoV-2 pandemic in Malawi had higher rates of severe maternal outcome, maternal death, and adverse neonatal outcomes than the fourth wave (omicron).

Previous literature comparing outcomes of COVID-19 in pregnancy associated with specific variants of concern include a study from the UK reporting data from periods of wild-type, alpha, and delta dominance,^22^ a single-centre study from India reporting data from periods of wild-type and delta dominance,^23^ and a study from Brazil reporting data from periods of wild-type and gamma (P.1) dominance.^24^ More recently, a retrospective study from the USA reported an unadjusted OR of 0·20 (95% CI 0·05–0·83) for severe or critical illness in pregnant women during a period of omicron dominance compared with a period before delta dominance,^12^ and a study from Mumbai, India reported an unadjusted OR of 0·2 (95% CI 0·1–0·3) of intensive or high dependency care being required in their omicron-associated wave compared with their delta-associated wave.^14^ To our knowledge, our study is the first to compare the clinical profiles and outcomes of COVID-19 in pregnancy across multiple waves of distinctive variant dominance in sub-Saharan Africa and is the first report of omicron-associated COVID-19 in maternity facilities in a low-resource setting, contributing significantly to the limited data available for omicron infection in pregnancy globally.

MATSurvey's surveillance and testing strategy found a SARS-CoV-2 positivity rate of 63% in pregnant and recently pregnant women admitted to health-care facilities in Malawi with symptoms of COVID-19. This rate was higher than in studies included in a recent systematic review update (search run in April, 2021; published in May, 2022), which found an overall symptomatic SARS-CoV-2 positivity rate of 22% (95% CI 12–33; 15 studies, 8128 women).^1^ Our findings probably reflect our study's high rates of testing (99%) and reporting (98%) for SARS-CoV-2 in patients admitted to hospital with symptoms of COVID-19, during a period of high incidence, supported by a significant increase in anti-SARS-CoV-2 antibodies within Malawi's population during the observation period (18% in October, 2020; 65% in May, 2021; and 70% in July, 2021 from retrospective blood bank seroprevalence data).^25^ It is also possible that resource limitations in our setting resulted in a high threshold for hospital admission; our participants might have had more severe disease and higher viral loads than inpatients in the majority of settings that have reported COVID-19 outcomes in pregnancy to date.

We found shortness of breath to be significantly less common in patients admitted to health-care facilities in Malawi in the fourth wave than in the second and third waves. Shortness of breath was also the only symptom associated with severe maternal outcome or maternal death. The better maternal outcomes observed during the fourth wave in Malawi are probably due to the omicron variant leading to less respiratory symptoms and failure.^26^, ^27^, ^28^ Although oxygen was available in all facilities included in our study, invasive ventilation was not available to patients with COVID-19, and continuous positive airway pressure was rarely available.

One of our study's strengths is its national geographical coverage, with clinical data submitted from all Central Hospitals, District Health Offices, and district hospitals in Malawi throughout three waves of the SARS-CoV-2 pandemic. The use of a tablet-based data entry approach resulted in few participants having missing data (5%, all in one data field only). To comprehensively record national burden, MATSurvey aimed to capture all maternal near-misses and maternal deaths in patients presenting to public hospitals in Malawi. In our population of admitted patients with proven symptomatic SARS-CoV-2 infection, the mortality rate was considerably high (17%), with a large difference between waves (second wave 25%, third wave 18%, fourth wave 5%). The high mortality in our cohort is consistent with a retrospective analysis published in 2022 that used data from the Democratic Republic of Congo, Ghana, Kenya, Nigeria, South Africa, and Uganda.^8^ This previous work found an increased risk of in-hospital death in pregnant women with SARS-CoV-2 compared with uninfected pregnant women (adjusted sub-hazard ratio 5·03 [95% CI 1·79–14·13]) and infected non-pregnant women (2·00 [1·08–3·70]). Reassuringly, the data for this retrospective analysis were collected before omicron emergence, and our findings suggest significantly lower maternal mortality during our observation period of omicron dominance.

The main limitation of our study is the use of periods of variants of concern dominance as a proxy for variants of concern-associated disease, given our study selected for patients admitted for care and we hypothesised that illness severity is increased for some variants of concern. Health-care infrastructure did not support national, comprehensive genomic coverage, nor prospective sampling from all patients admitted to hospital or those being managed in an outpatient setting. However, the signal from the fourth wave's genomic profile is suggestive of omicron being extensively dominant, with 100% of SARS-CoV-2 sequences from Malawi uploaded to GISAID in December, 2021 being the omicron variant.^20^

Additional limitations of our study include that it did not capture a number of important patient-level variables, including gestational age, mode of delivery, COVID-19 vaccine status, and COVID-19 therapeutics administered. Regarding the latter, our mixed-effects approach should have adjusted for any facility-level variations in medication availability, along with other differences in local clinical practice that might contribute to different maternal outcomes. In addition, our findings could be influenced by changes in practice and improvement in care of patients with COVID-19 within health-care facilities throughout the pandemic response, or due to changes in immunity. Cumulating vaccination coverage is unlikely to be responsible for our findings as, although severe COVID-19 in pregnancy has been shown to be generally restricted to unvaccinated patients,^29^ COVID-19 vaccination coverage was low during our observation period—at the beginning of the third and fourth waves, 1·9% and 6·0% of the population in Malawi (respectively) had received at least one COVID-19 vaccine (0% and 3·2% were fully vaccinated).^30^ Acquisition of natural immunity, however, could have contributed to our observations.^25^ Finally, our findings cannot be used to estimate the additional risk of adverse maternal or neonatal outcomes caused by each SARS-CoV-2 variant, as our population represented referrals from maternity services throughout the country. Outpatient symptomatic and asymptomatic rates of infection are not known so we cannot provide a reliable denominator. However, given that the maternal mortality ratio in Malawi is high (439 per 100 000 population in the latest Demographic and Health Survey)^31^ and that, before omicron emergence, pregnant and recently pregnant patients with COVID-19 have been found to have an odds of death 6·09 times greater than patients without,^1^ the burden of COVID-19 on pregnant and recently pregnant women in Malawi is likely to have been substantial. Future work is needed to establish the indirect effects of COVID-19 on maternity services in Malawi, as these have been shown to have substantial effects on maternal and child health services in other settings.^32^, ^33^, ^34^, ^35^

In summary, the fourth wave of the SARS-CoV-2 pandemic in Malawi, with omicron variant dominance, appeared to have lower rates of severe maternal outcome, maternal death, and neonatal adverse outcomes than the waves of beta and delta circulation that preceded. In the second and third waves of the SARS-CoV-2 pandemic in Malawi, with beta and delta variant dominance respectively, there was a profound burden of severe maternal outcomes, including maternal death, in our cohort of admitted pregnant and recently pregnant (≤42 days post-birth) patients with symptomatic SARS-CoV-2 infection.

Kuti muwelenge Chindunji cha kafukufuyi mu Chichewa, onani ku appendix

## Data sharing

The raw data used for this study cannot be publicly shared, as mandated within the College of Medicine Research Ethics Committee's approval, to ensure patient confidentiality. Requests for anonymised data, aggregated by facility, can be made by contacting the Malawi Ministry of Health and the Malawi–Liverpool–Wellcome Clinical Research Programme at dlissauer@mlw.mw.

## Declaration of interests

The Wellcome Trust has provided a Strategic Award to the Malawi–Liverpool–Wellcome Clinical Research Programme (206545/Z/17/Z) that, in part, covers the salary and operational costs of the Statistical Support Unit at the programme, led by MYRH. All other authors declare no competing interests.

## Supplementary Material

Chichewa translation of the abstract
