# Correction to *Lancet Glob Health* 2022; 11: e1623–31

**DOI:** 10.1016/j.langlo.2026.104056

**Published:** 2026-07-16

*Mndala L, Monk E J M, Phiri D, et al. Comparison of maternal and neonatal outcomes of COVID-19 before and after SARS-CoV-2 omicron emergence in maternity facilities in Malawi (MATSurvey): data from a national maternal surveillance platform.* Lancet Glob Health *2022;* ***11:*** *e1623–31*—In this Article, the second sentence of the Acknowledgement should have read “DL is supported by a National Institute for Health and Care Research (NIHR) Global Health Professorship (NIHR300808) and (NIHR134781), using UK aid from the UK Government to support global health research. The views expressed in this publication are those of the authors and not necessarily those of the NIHR or the UK Government”. This correction has been made as of July 9, 2026.

