# Supplementary material for: Comparison of maternal and neonatal outcomes of COVID-19 before and after SARS-CoV-2 omicron emergence in maternity facilities in Malawi (MATSurvey): data from a national maternal surveillance platform
Source: Lancet Glob Health. 2022 Sep 22;10(11):e1623–31. doi: 10.1016/S2214-109X(22)00359-X (PMC9553200; doi:10.1016/S2214-109X(22)00359-X)
Supplement: Chichewa translation of the abstract [file mmc1.pdf]

THE LANCET
Global Health

Supplementary appendix

This translation in Chichewa was submitted by the authors and we reproduce it as supplied. It has not been peer reviewed. *The Lancet*'s editorial processes have only been applied to the original in English, which should serve as reference for this manuscript.

Kutanthauzila kwa mu Chichewa uku kwachokela kwa alembi a nkhaniyi ndipo tayipeleka kwa inu m'mene tinayilandilila. Mawu a mu Chichewa sanawunikilidwenso kapena kukonzedwa. Nkhani ya mu Chingelezi yokha ndi imene yadutsa mu ukonzi wa Lancet, kotelo kuti nkhani ya mu Chingeleziyi ndi imene ikuyimilila mokwanila nkhani yonse imene yalembedwa.

Supplement to: Mndala L, Monk EJM, Phiri D, et al. Comparison of maternal and neonatal outcomes of COVID-19 before and after SARS-CoV-2 omicron emergence in maternity facilities in Malawi (MATSurvey): data from a national maternal surveillance platform. *Lancet Glob Health* 2022; published online Sept 22. https://doi.org/10.1016/S2214-109X(22)00359-X.

Kufananitsa zotsatira za uchembele wa amayi zobwela kamba ka COVID-19 mu nyengo ya omicron ndi nyengo ya tizilombo tina toyambitsa COVID-19: malipoti ochokela mu ndandanda wa amayi omwe adagonekedwa mu zipatala za m'dziko la Malawi (MATSurvey).

Chiyambi: Zotsatira zokhudza mlili wa matenda a COVID-19 odza kamba ka kachirombo ka omicron mwa amayi oyembekezera a m'maiko osauka sizidasindikidweko; ndipo malipoti ochokela mmaiko aku m'mwela kwa chipululu cha Sahara ku Africa, ndi ochepa. Pogwiritsa ntchito ma lipoti oyang'ana za zotsatira za COVID-19 mwa amayi amene adagonekedwa mu zipatala za m'dziko la Malawi (MatSurvey platform), tidapanga kafukufuku osiyanitsa zotsatira za kachirombo ka omicron ndi tizirombo tina tomwe tidavuta ku Malawi m'mbuyomu, ta beta ndi delta, mwa amayiwa.

Njira/Ndondomeko: Amayi onse oyembekezera, ndi ena omwe adali ndi mimba posachedwa ndipo abeleka kapena kupita padera, pasadathe masiku 42, ndi omwe adatenga nawo mbali mu kafukufufukuyu. Awa adali amayi okhawo omwe adagonekedwa m'zipatala 33 za boma ku Malawi, ndipo adapezeka ndi ka chirombo koyambitsa matenda a COVID-19 mu nyengo ya kachirombo ka beta [B.1.351] (January mpaka April 2021), ka delta [B.1.617.2] (June mpaka October 2021) ndi ka omicron [B.1.1.529] (December 2021 mpaka March 2022). Pogwiritsa ntchito njira ya ukadaulo ya Fischer's Exact Test, tidasiyanitsa zizindikiro za amayi, zotsatira za uchembele wawo, komanso zotsatira za ana obadwa mu nyengo ya tizirombo tosiyanasiyanati, toyambitsa matenda a COVID-19. Mulingo wa kusiyana kwa zotsatira za uchembele mwa amayiwa tidauyesa pogwiritsa ntchito njira ya mixed-effects logistic regression.

Zomwe tidapeza: Mu nyengo yakafukufukuyi (Jan 1, 2021 mpaka March 31, 2022), amayi 437 omwe adagonekedwa muzipatala 28 zaboma, ndi omwe adali ndi zizindikilo za COVID-19. Atayesedwa, amayi 261 ndi omwe zotsatira zawo zidatsimikiza kuti adali ndi matenda a COVID-19. Mwa amayiwa, 76 (29%) adakumana ndi mavuto aakulu odza chifukwa cha uchembele, pafupifupi kutaya miyoyo, ndipo 45 (17%) adamwalira. Mavuto akuluwa adali ochepa mu nyengo ya kachirombo ka omicron pofanizira ndi nyengo zina zomwe tizirombo ta beta (aOR 3.96, 95% CI 1.22–12.83, p=0.022) ndi delta (aOR 3.18, 95% CI 1.03–9.80, p=0.044) tidavuta ku Malawi kuno. Imfa zokhudzana ndi uchembele mwa amayi zidali zochuluka mu nyengo ya tizirombo ta beta (aOR 5.65, 95% CI 1.54–20.69, p=0.0090) ndi delta (aOR 3.52, 95% CI 0.98–12.60, p=0.053) pofanizira ndi mu nyengo ya omicron. Amayi ambiri omwe amabanika popuma ndi omwe adakumana ndi mavuto aakulu odza chifukwa cha uchembele (p<0.0001). Mu nyengo ya omicron, amayi ambiri omwe adagonekedwa mu zipatala sadali obanika nkapumidwe (23%) pofanizira ndi omwe adagonekedwa mu nyengo ya beta (51%; p=0.0007) ndi delta (50%; p=0.0004). Mbiri komanso mbiri ya zachipatala ya amayiwa idali yofanana mu nyengo ya tizirombo tosiyanasiyanati. Mu nyengo ya tizirombo ta beta ndi delta, ana 12 (13%) mwa ana 92 adamwalira kapena kubadwa okufa kale. Mwa ana omwe adabadwa mu nyengo ya omicron (25), padalibe olo ndi mmodzi yemwe (0) adamwalira kapena kubadwa okufa kale.

Tanthauzo/Ndemanga: Zotsatira za mlili wa matenda a COVID-19 mwa amayi oyembekezera, ndi ena omwe abeleka kapena kupita padera pasadathe masiku 42, zidali zochepelako mu nyengo ya kachirombo ka omicron pofanizira ndi nyengo yomwe tizirombo ta beta ndi delta tidasautsa muno m'Malawi.

Thandizo la ndalama: Ntchito ya kafukufukuyi idatheka ndi thandizo la ndalama lochokela ku bungwe la Bill and Melinda Gates, Wellcome Trust, ndi National Institute for Health and Care Research.
